# The Effect of Inhibitory Neuron on the Evolution Model of Higher-Order Coupling Neural Oscillator Population

**DOI:** 10.1155/2014/174274

**Published:** 2014-01-02

**Authors:** Yi Qi, Rubin Wang, Xianfa Jiao, Ying Du

**Affiliations:** ^1^Institute for Cognitive Neurodynamics, East China University of Science and Technology, Shanghai 200237, China; ^2^School of Mathematics, Hefei University of Technology, Hefei 230009, China

## Abstract

We proposed a higher-order coupling neural network model including the inhibitory neurons and examined the dynamical evolution of average number density and phase-neural coding under the spontaneous activity and external stimulating condition. The results indicated that increase of inhibitory coupling strength will cause decrease of average number density, whereas increase of excitatory coupling strength will cause increase of stable amplitude of average number density. Whether the neural oscillator population is able to enter the new synchronous oscillation or not is determined by excitatory and inhibitory coupling strength. In the presence of external stimulation, the evolution of the average number density is dependent upon the external stimulation and the coupling term in which the dominator will determine the final evolution.

## 1. Introduction

The quantitative analysis of the dynamics of neural oscillator population has become a highly publicized study [1, 2]. Gray and Singer found synchronous oscillations caused by external stimulus in cat primary visual cortex in 1989 [3]. Massive animal experiments suggested that the dynamics of synchronous oscillation is closely related to the message transition in the certain cortex [4].

The cerebral cortex is the unstable complicated nonlinear dynamical system. Furthermore, the nonlinear dynamics analysis method has succeeded applies in the neurodynamics research. The theory of phase transition dynamics was applied to the studies of physiological rhythms by Winfree [5] and had obtained lots of improvements by Kuramoto who described the dynamic evolution of neural oscillator population oscillation by the number density. P. A. Tass proposed theory of stochastic phase transition dynamics and further applied the theory triumphantly used to neurodegenerative disease [6–8]. Wang et al. applied stochastic dynamics of phase transformation to the research on cognitive neurodynamics and received many conclusions with actual physiological significance by means of numerical analysis and simulation [9–13]. The inhibitory neurons play an extremely important role in synchronous motion of neural oscillator populations and the evolution of neural coding [13]. Liu et al. proposed a stochastic nonlinear phase dynamic model under the coupling action of inhibitory neurons and analyzed the spontaneous behavior and the dynamic evolution of average number density under the condition of simulation. Trappenberg studied neural encoding and decoding of neural oscillator population under the strong inhibitory condition [14]. Weigenand et al. examined the phase response of cortical slow oscillation during deep sleep [15]. However, for easy analysis and processing, they studied the coupling between the neuronal oscillators mainly under the condition of lower-order coupling in most stochastic evolution models. Moreover, the actual synaptic connections between neurons are following nonlinear relationship and variable higher-order coupling. Zhang et al. studied the dynamical evolution of the phase-neural coding in the neural network under the condition of higher-order coupling. However, the function of inhibitory neurons in the neural coding evolution of neural oscillator populations is still unknown. In this paper, taking into account distribution of inhibitory neurons in the higher-order coupling condition, we proposed a new stochastic phase transition dynamics model and examined dynamical evolution of neural oscillator population under the effect of inhibitory neuron.

## 2. Dynamic Model

We assume that there are *N* neural oscillators in a neural network (*N* = *N*_1_ + *N*_2_), in which *N*_1_ excitatory neural oscillators are considered as a population and *N*_2_ inhibitory neural oscillator is another population:

(1) ψj•=Ω1+1N[∑k=1N1M11(ψj−ψk)       +∑k=N1+1NM21(ψj−ψk)] +S1(ψj)+Fj(t), j=1⋯N1,ψj•=Ω2+1N[∑k=1N1M12(ψj−ψk)       +∑k=N1+1NM2(ψj−ψk)] +S2(ψj)+Fj(t), j=N1+1⋯N,

where *ψ*_*j*_ is the phase of the oscillator number *j*; *Ω*_1_ and *Ω*_2_ are characteristic frequencies of excitatory and inhibitory neural oscillators population, respectively; *S*_1_(*ψ*_*j*_) and *S*_2_(*ψ*_*j*_) are the external stimulations on excitatory and inhibitory neural oscillators, respectively; *M*_11_(*ψ*_*j*_ − *ψ*_*k*_) is the mutual coupling interactions between excitatory coupling oscillators; and inhibitory coupling oscillators *M*_12_(*ψ*_*j*_ − *ψ*_*k*_), *M*_22_(*ψ*_*j*_ − *ψ*_*k*_), *M*_21_(*ψ*_*j*_ − *ψ*_*k*_)  *F*_*j*_(*t*) are Gaussian white noise, able to meet the following second-order condition:

(2) $$\begin{array}{c} \langle F_{j}\left(t\right)\rangle =0,\;\langle F_{j}\left(t\right)F_{k}\left(t^{\prime }\right)\rangle =Q\delta_{jk}\delta \left(t-t^{\prime }\right). \end{array}$$

Stimulation term is

(3) $$\begin{array}{c} S_{k}\left(\psi_{j}\right)=\overset{\infty }{\underset{m=1}{\sum }}I_{k_{m}}\cos\left(m\psi_{j}+\gamma_{k_{m}}\right),\;k=1,2. \end{array}$$

And coupling interactions between excitatory coupling oscillators and inhibitory coupling oscillators are

(4) M11(ψj−ψk)  =−∑m=14(Kmsin[m(ψj−ψk)]       +Cmcos[m(ψj−ψk)]),M12(ψj−ψk)  =−∑m=14(K1msin[m(ψj−ψk)]       +C1mcos[m(ψj−ψk)]),M22(ψj−ψk)  =−∑m=14(Lmsin[m(ψj−ψk)]       +Gmcos[m(ψj−ψk)]),M21(ψj−ψk)  =−∑m=14(L1msin[m(ψj−ψk)]       +G1mcos[m(ψj−ψk)]).

According to phase dynamic equation (1), we can get the Fokker-Planck equation of probability density *f*({*ψ*_*l*_}, *t*)

(5) ∂f({ψl},t)∂t=Q2∑j=1N∂2f({ψl},t)∂ψj2−∑j=1N1∂∂ψjΓ1f({ψl},t)−∑j=N1+1N∂∂ψjΓ2f({ψl},t),

where

(6) $$\begin{array}{c} \Gamma_{1}=\frac{1}{N}\overset{N}{\underset{k=1}{\sum }}\Gamma_{11},\;\;\Gamma_{2}=\frac{1}{N}\overset{N}{\underset{k=1}{\sum }}\Gamma_{22},\\ \Gamma_{11}=\{\begin{array}{cc} \Omega_{1}+M_{1}\left(\psi_{j}-\psi_{k}\right)+S_{1}\left(\psi_{j}\right), & k=1\cdots N_{1},\\ \Omega_{1}+M_{21}\left(\psi_{j}-\psi_{k}\right)+S_{1}\left(\psi_{j}\right), & k=N_{1}+1\cdots N, \end{array}\\ \Gamma_{22}=\{\begin{array}{cc} \Omega_{2}+M_{12}\left(\psi_{j}-\psi_{k}\right)+S_{2}\left(\psi_{j}\right), & k=1\cdots N_{1},\\ \Omega_{2}+M_{2}\left(\psi_{j}-\psi_{k}\right)+S_{2}\left(\psi_{j}\right), & k=N_{1}+1\cdots N. \end{array} \end{array}$$

The number density with the same phase *θ*_1_ of excitatory neural oscillator population and number density with the same phase *θ*_2_ of inhibitory neural oscillator population are described as:

(7) $$\begin{array}{c} {\tilde{n}}_{1}\left(\theta_{1}\right)=\frac{1}{N}\overset{N_{1}}{\underset{k=1}{\sum }}\delta \left(\theta_{1}-\psi_{K}\right),\\ {\tilde{n}}_{2}\left(\theta_{2}\right)=\frac{1}{N}\overset{N}{\underset{k=N_{1}+1}{\sum }}\delta \left(\theta_{2}-\psi_{K}\right). \end{array}$$

Considering the influence of stochastic noise, average number density of excitatory and inhibitory neural oscillator population is introduced as

(8) $$\begin{array}{c} n_{1}\left(\theta_{1},t\right)=\overset{2\pi }{\underset{0}{\int }}\cdots \overset{2\pi }{\underset{0}{\int }}d\psi_{1}\cdots d\psi_{N}{\tilde{n}}_{1}f,\\ n_{2}\left(\theta_{2},t\right)=\overset{2\pi }{\underset{0}{\int }}\cdots \overset{2\pi }{\underset{0}{\int }}d\psi_{1}\cdots d\psi_{N}{\tilde{n}}_{2}f. \end{array}$$

Global number density of neural oscillators is described as:

(9) $$\begin{array}{c} n\left(\theta_{1},\theta_{2},t\right)=n_{1}\left(\theta_{1},t\right)+n_{2}\left(\theta_{2},t\right). \end{array}$$

The above number density under following boundary condition

(10) $$\begin{array}{c} \overset{2\pi }{\underset{0}{\int }}n_{1}\left(\theta_{1},t\right)d\theta_{1}=\frac{N_{1}}{N},\;\;\overset{2\pi }{\underset{0}{\int }}n_{2}\left(\theta_{2},t\right)d\theta_{2}=\frac{N-N_{1}}{N},\\ n_{1}\left(0,t\right)=n_{1}\left(2\pi ,t\right),\;\;n_{2}\left(0,t\right)=n_{2}\left(2\pi ,t\right). \end{array}$$

Taking into account (5), (7), and (8), one obtains

(11) ∂n∂t=−∂∂θ1{n1(θ1,t)∫02πdψ′[M1(θ1−ψ′)n1(ψ′,t)        +M21(θ1−ψ′)n2(ψ′,t)]}  −∂∂θ2{n2(θ2,t)∫02πdψ′[M12(θ2−ψ′)n1(ψ′,t)        +M2(θ2−ψ′)n2(ψ′,t)]}  −(∂∂θ1n1(θ1,t)S1(θ1)+∂∂θ2n2(θ2,t)S2(θ2))  −(Ω1∂∂θ1n1(θ1,t)+Ω2∂∂θ2n2(θ2,t))  +Q2(∂2n1(θ1,t)∂θ12+∂2n2(θ2,t)∂θ22).

Decomposition of (11), equation expressing number density evolution obtains

(12) ∂n1(θ1,t)∂t =Q2∂2n1(θ1,t)∂θ12−Ω1∂∂θ1n1(θ1,t)  −∂∂θ1n1(θ1,t)S1(θ1)  −∂∂θ1{n1(θ1,t)∫02πdψ′[M1(θ1−ψ′)n1(ψ′,t)                +M21(θ1−ψ′)n2(ψ′,t)]},∂n2(θ2,t)∂t =Q2∂2n2(θ2,t)∂θ22−Ω2∂∂θ2n2(θ2,t)  −∂∂θ2n2(θ2,t)S2(θ2)  −∂∂θ2{n2(θ2,t)∫02πdψ′[M12(θ2−ψ′)n1(ψ′,t)              +M2(θ2−ψ′)n2(ψ′,t)]}.

## 3. Numerical Analysis 

### 3.1. The Effect of the Higher-Order Coupling Term in Inducing the Synchronization of the Inhibitory Neuronal Oscillator Population under Spontaneous Activity

In the case of spontaneous activity, in order to study the effects of different order of higher-order coupling on neural oscillator population, we set coupling terms as *C*_*m*_ = 0, *C*_1*m*_ = 0, *G*_*m*_ = 0, *G*_1*m*_ = 0, and *γ*_*k*_*m*__ = 0 in (4). *K*_*m*_, *K*_1*m*_, *L*_*m*_, and *L*_1*m*_ are constant-coefficient coupling terms of *m*-order coupling. The constant-coefficient coupling terms of excitatory coupling are plus and constant-coefficient coupling terms of inhibitory coupling are minus [9], *K*_*m*_ > 0, *K*_1*m*_ > 0, *L*_*m*_ < 0, *L*_1*m*_ < 0. And coupling term is described as

(13) $$\begin{array}{c} M_{1}\left(\psi_{j}-\psi_{k}\right)=-K_{3}\sin\left\lfloor 3\left(\psi_{j}-\psi_{k}\right)\right\rfloor ,\\ M_{12}\left(\psi_{j}-\psi_{k}\right)=-K_{13}\sin\left[3\left(\psi_{j}-\psi_{k}\right)\right],\\ M_{2}\left(\psi_{j}-\psi_{k}\right)=-L_{3}\sin\left[3\left(\psi_{j}-\psi_{k}\right)\right],\\ M_{21}\left(\psi_{j}-\psi_{k}\right)=-L_{13}\sin\left[3\left(\psi_{j}-\psi_{k}\right)\right]. \end{array}$$

The initial conditions were chosen as

(14) $$\begin{array}{c} n_{1}\left(\psi ,0\right)=\frac{N_{1}}{N}\left(\frac{1}{2\pi }+0.5\sin\left(\psi \right)\right),\\ n_{2}\left(\psi ,0\right)=\frac{N-N_{1}}{N}\left(\frac{1}{2\pi }+0.5\sin\left(\psi \right)\right). \end{array}$$

#### 3.1.1. The Impact of Inhibitory Coupling on Synchronous Activity of Neural Oscillator Population

**Figure 1:**
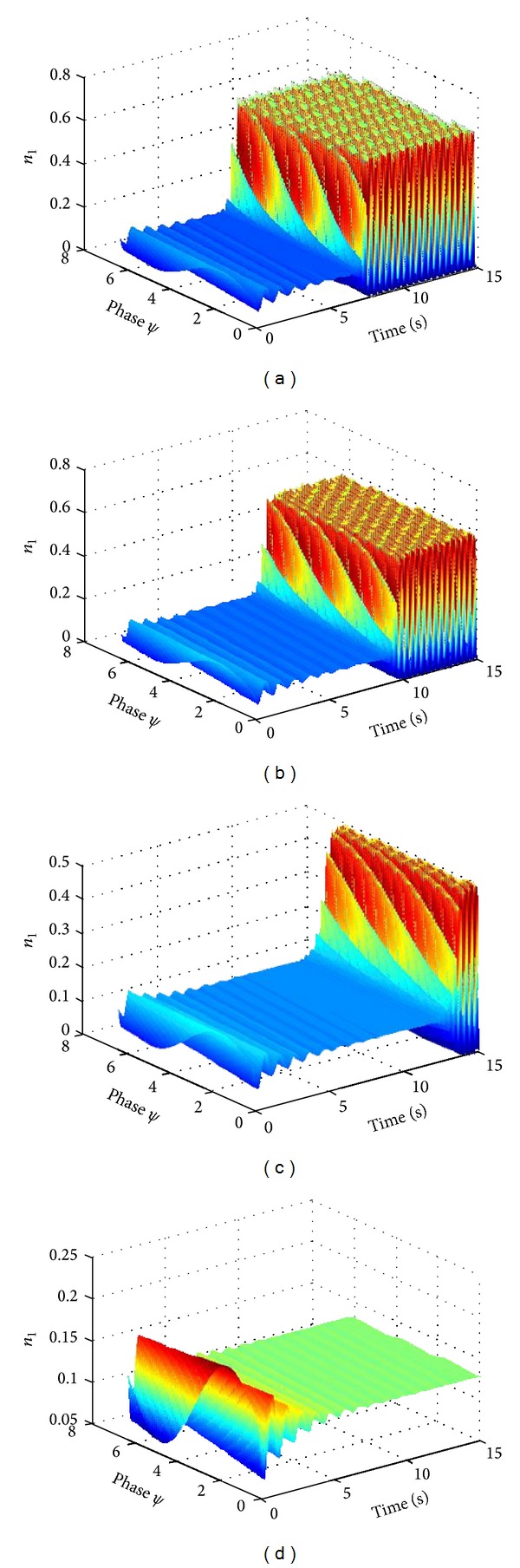
The evolution of the average number density of neural oscillator population with respect to variable inhibitory coupling strength. *N*_1_ = 800, *N*_2_ = 200, *N* = 1000, *K*_3_ = 8, *K*_13_ = 8, (a) *L*_3_ = 0, *L*_13_ = 0, (b) *L*_3_ = −4, *L*_13_ = −4, (c) *L*_3_ = −8, *L*_13_ = −8, (d) *L*_3_ = −10, *L*_13_ = −10.

In order to study the impact of inhibitory coupling on synchronous activity of neural oscillator population, we assumed that there is a third-order coupling between excitatory neural oscillators, and the coupling strength is described as *K*_3_ = *K*_13_ = 8. The detention of synchronous activity of neural oscillator population was induced by inhibitory coupling (Figure 1(b)). And the synchronization state of neural oscillator population decreased along with increase in inhibitory coupling strength (Figure 1(c)). The neural oscillator population rapidly underwent a process from initial synchronization state entering into desynchronization state when the coupling intensity increases to certain degree (Figure 1(d)).

We assumed that the neurons reach action potentials immediately when the phase *ψ* = 0. According to this assumption, we can describe firing density of neural oscillator population as *p*(*t*) = *n*_1_(0, *t*) and estimate the cluster number of neural oscillator population in which fire action potential simultaneously by using of *p*(*t*).

**Figure 2:**
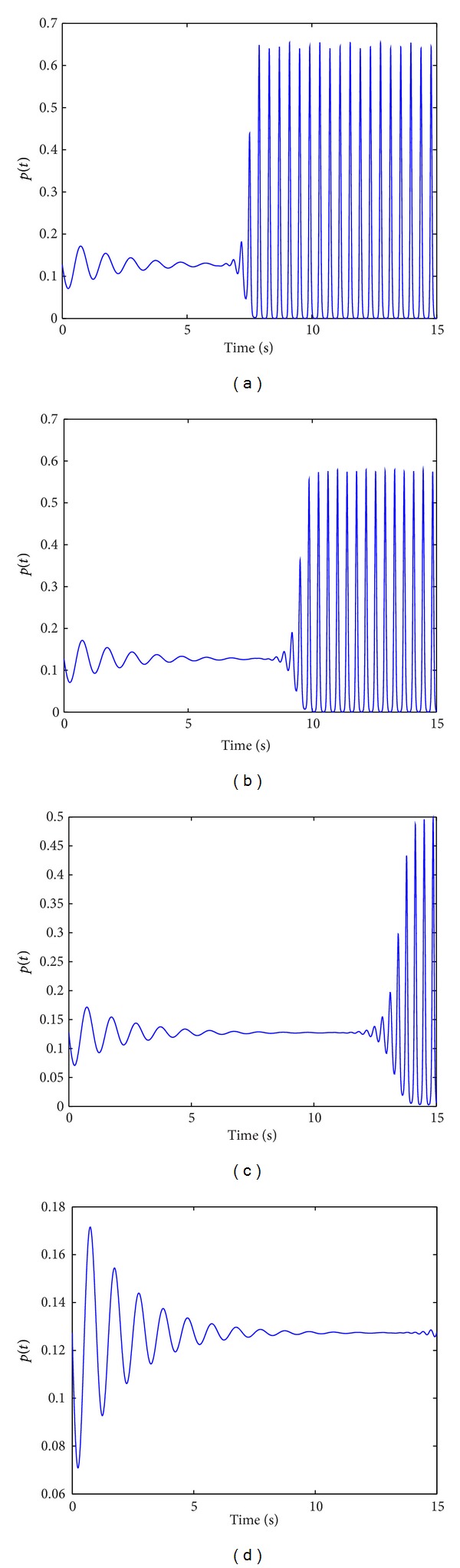
The evolution of firing density with respect to variable inhibitory coupling strength with increasing time. *p*(*t*) = *n*_1_(0, *t*), *N*_1_ = 800, *N*_2_ = 200, *N* = 1000, *K*_3_ = 8, *K*_13_ = 8, (a) *L*_3_ = 0, *L*_13_ = 0, (b) *L*_3_ = −4, *L*_13_ = −4, (c) *L*_3_ = −8, *L*_13_ = −8, (d) *L*_3_ = −10, *L*_13_ = −10.

Figure 2 illustrated the firing density evolution of neural oscillator population with increasing time. In the absence of inhibitory coupling, the firing density reaches the peak, which means the most neural oscillator population firing action potential (Figure 2(a)). The inhibitory coupling delays the synchronous activity of neural oscillator population and synchronous activity gradually decays as the inhibitory coupling strength increases (Figures 2(b) and 2(c)). This result indicated that decrease of cluster number with synchronous activity is induced by increase of inhibitory coupling strength. The neuronal oscillator population will enter a desynchronization state when the inhibitory coupling reaches the certain strength (Figure 2(d)).

#### 3.1.2. The Impact of Excitatory Coupling on Synchronous Activity of Neural Oscillator Population

**Figure 3:**
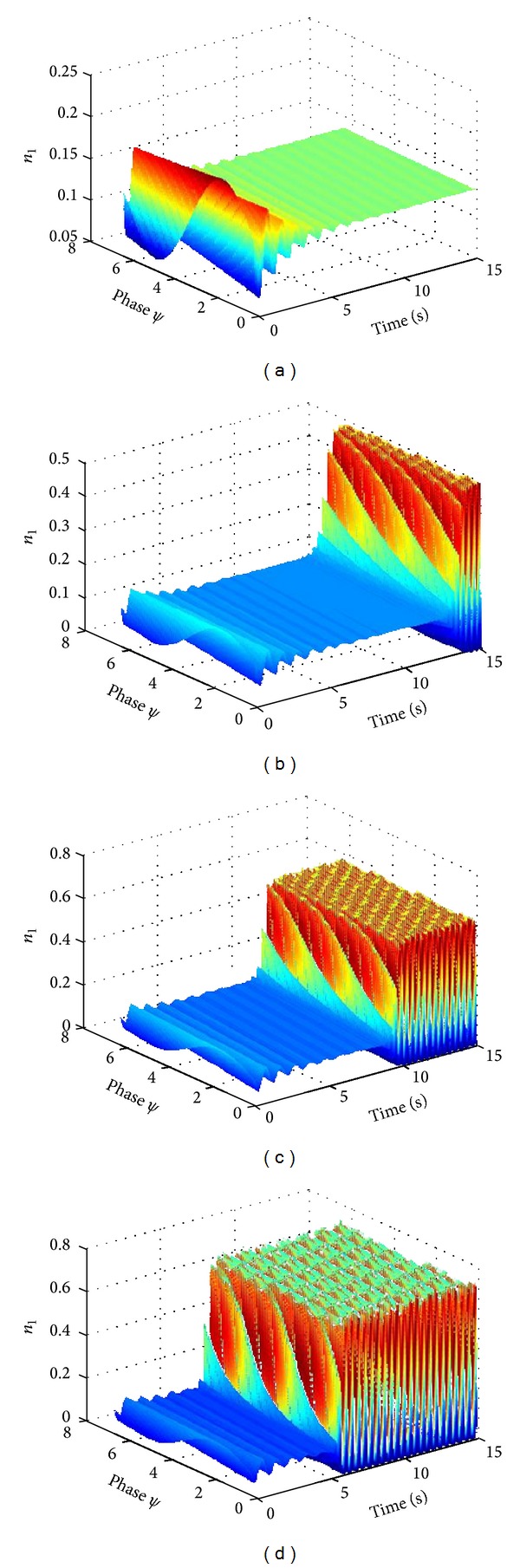
The evolution of the average number density of neural oscillator population with respect to variable excitatory coupling strength. *N*_1_ = 800, *N*_2_ = 200, *N* = 1000, *L*_3_ = −4, *L*_13_ = −4, (a) *K*_3_ = *K*_13_ = 4, (b) *K*_3_ = *K*_13_ = 7, (c) *K*_3_ = *K*_13_ = 8, (d) *K*_3_ = *K*_13_ = 10.

In order to study the impact of inhibitory coupling on synchronous activity of neural oscillator population, we assumed that the inhibitory coupling strength remains unchanged and is described as *L*_3_ = *L*_13_ = −4 in third-order coupling condition. The average number density gradually decays until evenly distributed as the excitatory coupling strength is described as *K*_3_ = *K*_13_ = −4, which indicates a desynchronization state of neural oscillator population. The neural oscillator population rapidly undergoes a process from initial desynchronization state entering into synchronization state when the excitatory coupling strength is described as *K*_3_ = *K*_13_ = 7 (Figure 3(b)). And the neural oscillator population rapidly entered a synchronization state along with increase in excitatory coupling strength (Figures 3(b), 3(c), and 3(d)).

**Figure 4:**
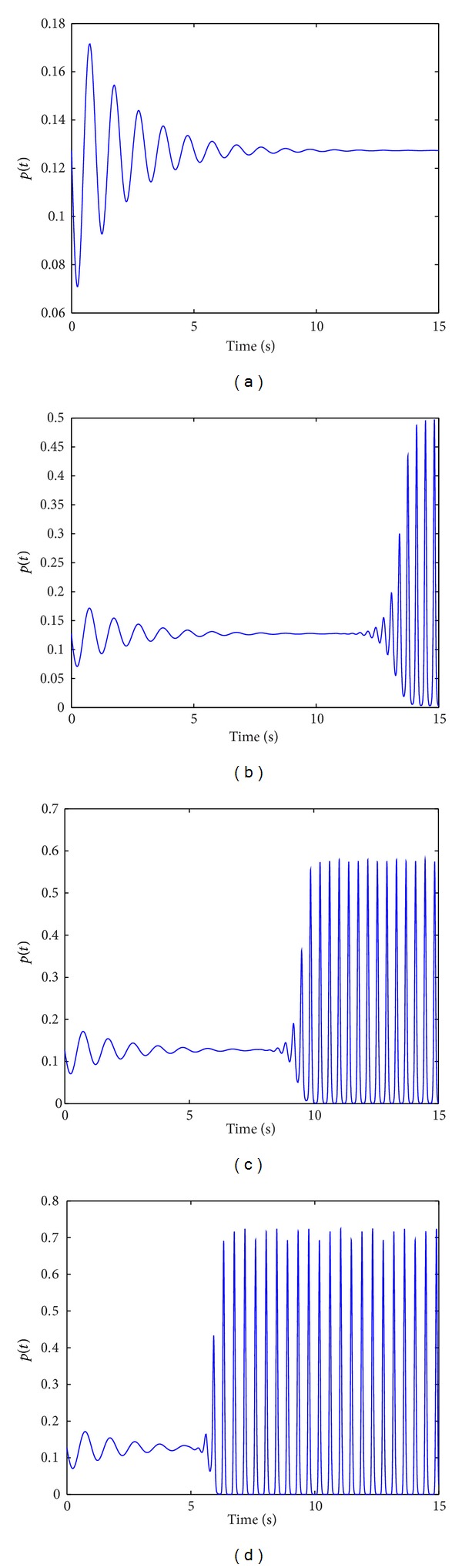
The evolution of firing density with respect to variable excitatory coupling strength with increasing time. *p*(*t*) = *n*_1_(0, *t*), *N*_1_ = 800, *N*_2_ = 200, *N* = 1000, *L*_3_ = −4, *L*_13_ = −4, (a) *K*_3_ = *K*_13_ = 4, (b) *K*_3_ = *K*_13_ = 7, (c) *K*_3_ = *K*_13_ = 8, (d) *K*_3_ = *K*_13_ = 10.

Figure 4 illustrated the firing density evolution of neural oscillator population with increasing time. The firing density gradually decays to an even distribution with increasing time (Figure 4(a)). These results confirm that the stronger the coupling strength is, the higher the firing density of neural oscillator population is, and the more cluster firing action potential is (Figures 4(b), 4(c), and 4(d)). This result indicated that increase of cluster number with synchronous activity is induced by increase of excitatory coupling strength. And the shorter time required for the neural oscillator population entering a new synchronous oscillation when the excitatory coupling strength increases (Figures 4(b), 4(c), and 4(d)).

### 3.2. The Impact of Coupling Order on Firing Density of Action Potential *p*(*t*)

**Figure 5:**
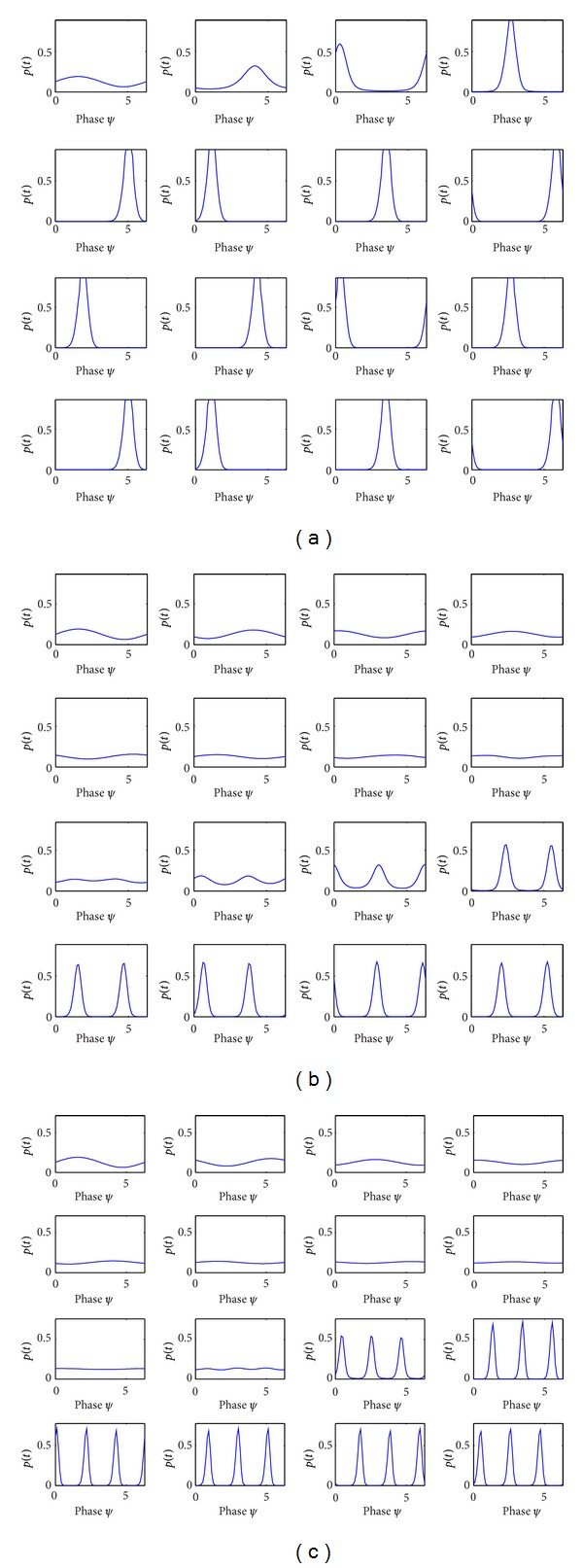
(a) Firing density evolution diagram of neural oscillators population with first-order coupling term. *N*_1_ = 800, *N*_2_ = 200, *N* = 1000, *K*_1_ = 8, *K*_11_ = 8, (a) *L*_1_ = −3, *L*_11_ = −3. (b) Firing density evolution diagram of neutral oscillators population with second-order coupling term. *N*_1_ = 800, *N*_2_ = 200, *N* = 1000, *K*_2_ = 8, *K*_12_ = 8, (a) *L*_2_ = −4, *L*_12_ = −4. (c) Firing density evolution diagram of neutral oscillators population with third-order coupling term. *N*_1_ = 800, *N*_2_ = 200, *N* = 1000, *K*_3_ = 8, *K*_13_ = 8, *L*_3_ = −4, *L*_13_ = −4.

Firing density evolution diagram of neural oscillator population is determined by the coupling term as shown in Figures 5(a), 5(b), and 5(c). The evolution of firing action potential density *p*(*t*) has only one peak with time under first-order coupling condition, which suggests one cluster of neuronal oscillators firing action potential at certain phase space. The evolution of firing density with increasing time in the second-order coupling term has been turned into the distribution of two peaks from that of one peak in the initial condition, which means two clusters of neuronal oscillators firing action potential. Three peaks for the firing density that appeared in the third-order coupling term showed the phase transition process of the neural oscillator population from one-cluster synchronization state in the initial condition to three-cluster synchronization state. Taken together, the synchronized firing density of neural oscillator population is determined by the order of neural coupling.

### 3.3. The Impact of External Stimulation on the Phase Coding of Inhibitory Higher-Order Coupling Neural Oscillator Population

In order to study the dynamical evolution of the average number density of neuronal oscillator with inhibitory coupling in presence of external stimulation, we selected the initial condition as

(15) $$\begin{array}{c} n_{1}\left(\psi ,0\right)=\frac{N_{1}}{N}\left(\frac{1}{2\pi }+0.5\sin\left(\psi \right)\right),\\ n_{2}\left(\psi ,0\right)=\frac{N-N_{1}}{N}\left(\frac{1}{2\pi }+0.5\sin\left(\psi \right)\right). \end{array}$$

Taking into account third-order coupling term

(16) $$\begin{array}{c} M_{1}\left(\psi_{j}-\psi_{k}\right)=-K_{3}\sin\left\lfloor 3\left(\psi_{j}-\psi_{k}\right)\right\rfloor ,\\ M_{12}\left(\psi_{j}-\psi_{k}\right)=-K_{13}\sin\left[3\left(\psi_{j}-\psi_{k}\right)\right],\\ M_{2}\left(\psi_{j}-\psi_{k}\right)=-L_{3}\sin\left[3\left(\psi_{j}-\psi_{k}\right)\right],\\ M_{21}\left(\psi_{j}-\psi_{k}\right)=-L_{13}\sin\left[3\left(\psi_{j}-\psi_{k}\right)\right]. \end{array}$$

And constant-coefficient coupling term is *K*_3_ = *K*_13_ = *L*_3_ = *L*_13_ = −4.

Taking into account second-order term

(17) $$\begin{array}{c} S_{k}\left(\psi_{j}\right)=I_{k1}\cos\left(\psi_{j}+\gamma_{k1}\right)+I_{k2}\cos\left(2\psi_{j}+\gamma_{k2}\right),\;k=1,2. \end{array}$$

**Figure 6:**
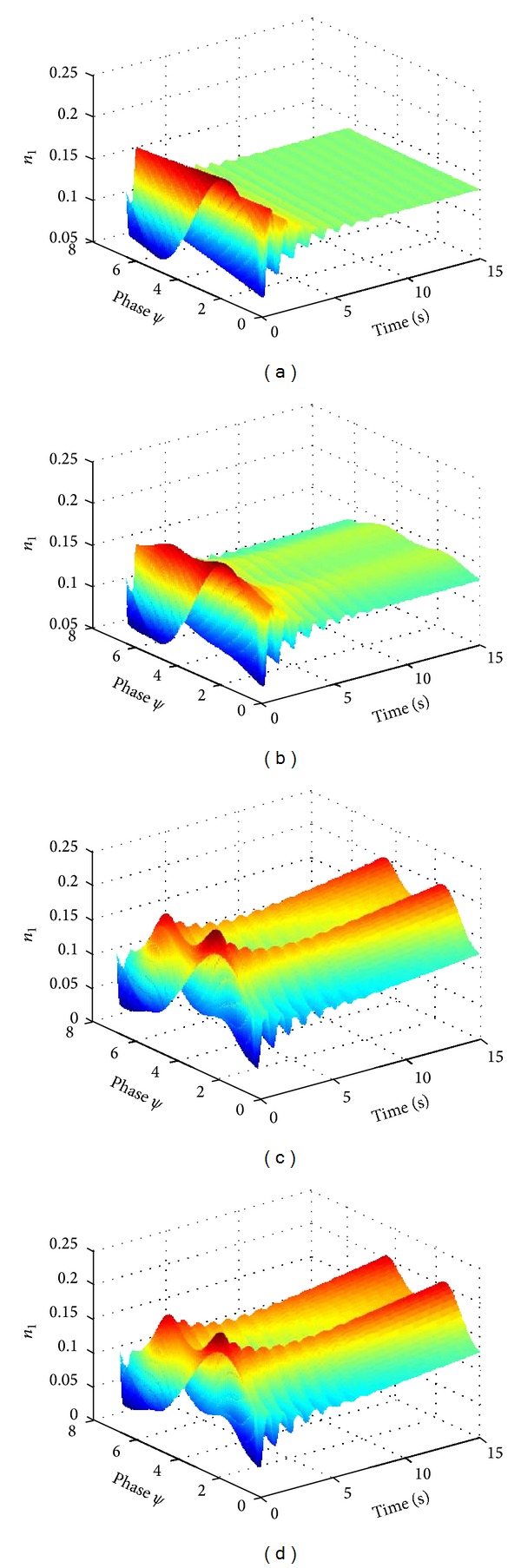
The impact of second-order stimulation on synchronous activity of neural oscillator population of inhibitory coupling. (a) *I*_11_ = *I*_12_ = *I*_21_ = *I*_22_ = 0; (b) *I*_11_ = *I*_12_ = *I*_21_ = *I*_22_ = 0.2; (c) *I*_11_ = *I*_12_ = *I*_21_ = *I*_22_ = 1; (d) noncoupling state *I*_11_ = 1, *I*_21_ = 1.

In case of no stimulation, the average number density of neural oscillator population gradually decays to totally scattered, which means that neural oscillator population enters into a desynchronization state (Figure 6(a)). The neural oscillator population will gradually turn into a full synchronization state along with increasing stimulation intensity (Figures 6(b) and 6(c)).

Figure 6(d) showed the evolution of neural oscillator population in the presence of external stimulation if the coupling strength is zero. We found that the evolution of neural oscillator population is very similar between Figures 6(c) and 6(d). The dynamical evolution of neural oscillator population is almost the same between coupling and noncoupling state, which suggested that the intensity of the external stimulation is the dominated factor for the dynamical evolution.

## 4. Conclusion

1. In case of spontaneous activity of neurons, the amplitude of the average number density evolution tends to decrease if the inhibitory coupling strength increases. In contrast, the amplitude of the average number density evolution tends to increase if the excitatory coupling strength increases.
2. In the case of stable excitatory coupling strength, synchronous activity of neuronal oscillator population gradually decays until it loses synchronous oscillation as the inhibitory coupling strength increases. On the other hand, in the case of stable inhibitory coupling strength, increase of excitatory coupling strength will result in enhancement of synchronous activity of neuronal oscillator population and then in it entering a new synchronization state.
3. Diagram of the firing density of action potential *p*(*t*) is determined by the order of coupling, when the neuronal oscillator population enters a new synchronization state at certain coupling strength. *N* peaks for the firing density appeared in the *n*-order coupling term which suggests neuronal oscillators firing action potential at *n*-certain phase space.
4. In the presence of external stimulation, the evolution of the average number density is dependent upon the stimulation and the neural structure of the coupling in which the dominator will decide the final evolution.
